# Leader Humility and Knowledge Sharing Intention: A Serial Mediation Model

**DOI:** 10.3389/fpsyg.2020.560704

**Published:** 2020-12-08

**Authors:** Diep T. N. Nguyen, Stephen T. T. Teo, Beni Halvorsen, Warren Staples

**Affiliations:** ^1^School of Business and Law, Edith Cowan University, Joondalup, WA, Australia; ^2^School of Management, RMIT University, Melbourne, VIC, Australia

**Keywords:** leader humility, affective trust, work engagement, OCB, knowledge sharing, social exchange theory

## Abstract

**Purpose:**

This paper examines the influence of leader humility on knowledge sharing intention. Drawing on social exchange theory (SET), we test the direct and indirect mechanisms to explain the influence leader humility has on knowledge sharing intention.

**Design/Methodology/Approach:**

A two-wave, time-lagged field study was conducted. We surveyed 252 professional employees from Australia.

**Findings:**

Results show a significant direct, positive association between leader humility and knowledge sharing intention. While leader humility had a direct, positive association with affective trust in supervisor and work engagement, it did not directly impact on organizational citizenship behaviors directed toward the individual (OCB-I). There were three SET-related, serial mediators in the relationship between leader humility and knowledge sharing intention. These were affective trust, work engagement, and OCB-I.

**Research Limitations/Implications:**

Future studies should collect multi-source data such as peers’ or supervisors’ ratings of the focal respondents’ work engagement, OCB-I, and knowledge sharing behaviors to augment single-source data. Future studies could adopt an affect theory of social exchange to further explore the relationships tested in this study.

**Originality/Value:**

This study contributes to the affect SET and knowledge management literature on how leadership behaviors impact the intention to share knowledge. Our study highlights the preference of the willingness to share knowledge with their co-workers is mediated by affective trust in their immediate supervisors, work engagement, and OCB-I that are equally important as treating their subordinates with humility.

## Introduction

Knowledge is a critical source of sustainable competitive advantage (Spender and Grant, 1996), and organizational success relies heavily on employees’ motivation and willingness to share knowledge with others (Wang and Noe, 2010). Knowledge sharing is “the act of making knowledge available to others within the organizations” (Ipe, 2003, p. 32) and is dependent on the behavioral intention of employees (see Gagné, 2009). Gagné (2009) examined the influences of human resource management (HRM) practices on knowledge sharing and proposed a framework of knowledge-sharing motivation. Wang and Noe’s (2010) extended Gagné’s (2009) framework to include organizational-level factors such as leadership behaviors, interpersonal relationships, and their influences on knowledge sharing.

The two widely employed theoretical frameworks in the research on knowledge sharing are self-determination theory (Gagné, 2009) and theory of planned behavior (TPB; Wang et al., 2015; Stenius et al., 2017). Meanwhile, social exchange theory (SET; Blau, 1964) has not received much attention in the knowledge management literature (Wang and Noe, 2010). Knowledge sharing intention can be theorized as an outcome of high-quality social exchange relationships at work (Bock et al., 2005; Wang et al., 2015). Scholars (Wang and Noe, 2010; Gagné et al., 2019) have called for more research to examine the direct and indirect role of leadership in promoting a trustworthy social exchange environment to motivate knowledge sharing among employees.

Serenko and Bontis (2016) contended that positive interpersonal relationships among employees that focused on “common good” instead of reciprocal benefits could facilitate the willingness of knowledge sharing. We argued that leadership is critical to stimulate positive interpersonal relationships concerning knowledge sharing (Le and Lei, 2018). An under-research concept in the knowledge sharing literature is leader humility (Anand et al., 2019). Leader humility is defined as a set of interpersonal qualities consist of “(a) a manifested willingness to view oneself accurately, (b) a displayed appreciation of others’ strengths and contributions, and (c) teachability, or openness to new ideas and feedback” (Owens et al., 2013, p. 1518). Our study adopts this definition of leader humility to explain employees’ knowledge sharing intention (Stenius et al., 2017; Yang et al., 2019) can be enhanced with the inclusion of affective trust in supervisor, one of the most vital factors influencing knowledge sharing intention and behaviors (see Navimipour and Charband, 2016), work engagement, and organizational citizenship behaviors directed toward the individual (OCB-I; Nielsen and Marrone, 2018). These three SET-related factors will contribute to our understanding of the mediation processes between leader humility and knowledge sharing intention. Building on Wang et al.’s (2015) research on how leader humility influences employees’ knowledge sharing intention, our study makes two main contributions. First, we apply Blau’s SET (Blau, 1964; Cropanzano and Mitchell, 2005) to examine the mediation mechanism underlying the relationship between leader humility and knowledge sharing intention. Second, we investigate three sequential exchange-based mediators: affective trust, work engagement, and OCB-I, as we expect humble leaders to create the social exchange relationship that stimulates knowledge sharing intention as obligation and reciprocity of their subordinates. To the best of our knowledge, there has not been any empirical study which considers the serial mediation of these social exchange variables in knowledge sharing.

## Literature Review and Hypotheses

### SET, Leader Humility, and Knowledge Sharing

Social exchange theory could be used to explain the influence of leadership and trust play in encouraging employees to share knowledge (Navimipour and Charband, 2016; Gagné et al., 2019). There is a scarcity of research uncovering the mechanisms showing how leadership behaviors influence knowledge sharing (Wang and Noe, 2010; Gerpott et al., 2019). Leaders who exhibit humility behaviors can motivate their employees to engage in positive behaviors (Owens and Hekman, 2016). A humble leader is someone who does not possess negative traits, such as arrogance, and is likely to provide an opportunity for employees to give opinions and raise concerns at work (Argandona, 2015). Also, they are more willing to listen to followers, accept opposite feedback, and take accountability for her/his mistakes and failures. Humble leaders are likely to share success with their subordinates, express gratitude, appreciation, and recognize their achievements (Argandona, 2015). These qualities can contribute to the creation of reciprocal attitudes and behaviors through a social exchange process.

Social exchange theory posits that the organizational system shapes social connections and interactions between people (Cropanzano and Mitchell, 2005). Indeed, high-quality interdependent relationships with leaders will generate employees’ obligations and commitment as they understand how they should reciprocate in mutual, respectful, and complementary activities (Cropanzano et al., 2017). “Subordinates may change their attitudes or behaviors, depending on how they perceive they are being treated or on the need for reciprocity” (Kim et al., 2015, p. 603).

A humble leader is likely to create a collaborative environment in which employees are encouraged to cooperate with others and be open to discussing and sharing opinions and ideas in solving problems (Owens et al., 2013; Anand et al., 2019). A humble leader should then provide the support necessary for their subordinates to generate the willingness to embrace new ideas, exchange information, and value individual contributions that promote proactive and collaborative interpersonal relationships among employees (Owens and Hekman, 2016). Based on reciprocal norms, employees develop a sense of obligation to collaborate with others and share knowledge (Bartol et al., 2009; Kim et al., 2015). Knowledge sharing allows organizations to exploit and capitalize on knowledge-based resources (Cabrera and Cabrera, 2005). Consistent with SET, employees may decide whether to engage in knowledge sharing behaviors depending on how their supervisors treat them at work (Kim et al., 2015). Therefore, we expect leader humility to create social workplace relationships for employees to share knowledge. We hypothesize that:

Hypothesis 1: There is a positive association between leader humility and employee’s knowledge sharing intention.

### Leader Humility and Affective Trust

While there is an increasing focus on understanding the consequences of leader humility, its impact on trust has not received much attention (Owens et al., 2013; Anand et al., 2019). As SET postulates, employees will adjust their attitudes and behaviors in correspondence with the treatment they receive (Cropanzano et al., 2017). To encourage knowledge sharing, factors such as organizational (in this case, leader humility) and motivational (trust) are essential (Wang and Noe, 2010). Knowledge sharing literature shows trusting relationships between individuals are crucial in creating the environment necessary for individuals’ willingness to share knowledge (Wang and Noe, 2010; Schein, 2013; Anand et al., 2019). Other scholars argue that leaders must first establish high-quality relationships with their subordinates in exchange for reciprocal behaviors (Wu and Lee, 2017) through subordinates’ affective trust toward their supervisors (Zhang and Jiang, 2015).

McAllister (1995) conceptualized two types of trust: cognitive trust and affective trust, which influence the attitudinal and behavioral responses of employees to the perceived relationship with their supervisors. The former dimension refers to employees’ objective assessment or personal appraisal of the leader’s ability, competence, integrity, and reliability. The latter factor refers to the emotional bonds the employees have developed with the leader, who reciprocate their exhibition of care and concern for each other’s well-being (Dirks and Ferrin, 2002). Affective trust is an outcome of the engagement of both employees and supervisors in a social exchange process to gain mutual concern and care for each other (Yang and Mossholder, 2010).

We were interested in affective trust because of several reasons. First, a humble leader is likely to provide individualized support and motivation to the subordinates by listening to employees, being fair, honest, and opened to opinions, and recognizing individual strengths and contributions (Owens et al., 2013; Owens and Hekman, 2016). This relationship with leader humility results in social cues that initiate a tight relational bond between supervisors and employees as employees feel respected, safe, appreciated, recognized, and fairly treated (Owens and Hekman, 2016), eliciting higher affective trust toward supervisors. Second, a humble leader is willing to involve employees in decision-making and listen to their advice and feedback that builds up strong interpersonal relationships with subordinates that go beyond the social exchange. Next, the self-disclosure element of leader humility (accepting one’s limitations) that is not the impression management strategy of the leader (Yang et al., 2019) signals employees that the supervisor cares for employees’ feelings and opinions so they can trust the leader in sharing information and receiving feedback (Bharanitharan et al., 2019). Leader humility also shows vulnerability with employees and shapes their perceptions of a power-equalized workplace that enables a sense of trust in leaders (Owens and Hekman, 2012; Wang et al., 2018; Yang et al., 2019). This relationship makes employees reciprocate positive attitudes and behavior such as work engagement, in line with the focus on the exchange of socio-emotional benefits between supervisors and employees. Wang et al. (2019) provided additional support for the positive association between leader humility and employees’ trust in their supervisors as employees reciprocate with beneficial and positive behaviors. Finally, Zhu et al. (2013) found that affective trust had positive relationships with affective organizational commitment, OC, and job performance, supporting our study’s SET perspective that highlights the mutual exchange of cooperation, concern, and care between employees and supervisors. We hypothesize the following:

Hypothesis 2a: There is a positive association between leader humility and employees’ affective trust in their supervisor.

### Leader Humility, Work Engagement, and OCB-I

Social exchange theory can also be used to explain employees’ engagement at work. Social exchange theory theory proposes that workplace relationships are built around mutual obligations. Accordingly, when a leader treats employees with respectful and fair treatment, this relationship will lead to a positive workplace experience (Cropanzano et al., 2017). Work engagement is one such positive workplace experience (Saks, 2006; Bakker, 2017). When employees are engaged with their work, this will generate positive feelings of energy, devotion, and fascination (Schaufeli et al., 2002). Work engagement is a “motivational concept” (Rich et al., 2010, p. 619). An engaged employee is someone who has a high level of emotional resilience, full of energy, perform their jobs happily and enthusiastically, and maintain positive perceptions of dedication and satisfaction with their tasks (Schaufeli and Bakker, 2004).

Humble leaders provide employees with an opportunity to learn about their strengths and weaknesses to improve their capabilities (Owens et al., 2013; Nielsen and Marrone, 2018). Additionally, a humble leader communicates and gives feedback to employees through non-judgmental, open, and candid communication such that employees feel free to share new ideas (Owens et al., 2013). Such support makes employees feel more engaged at work (Nielsen and Marrone, 2018). Other empirical evidence shows that leader humility helps employees to reduce stress or emotional exhaustion by increasing employee energy to take more efforts in performing their tasks and engaging at work (Owens and Hekman, 2012; Owens et al., 2016; Wang et al., 2018). We hypothesize that

Hypothesis 2b: There is a positive association between leader humility and employees’ work engagement.

Leader humility also plays a critical role in understanding employee’s citizenship behaviors (Nielsen and Marrone, 2018). As we argued, a humble leader becomes a role model showing their care for employees’ psychological needs and well-being (Owens et al., 2013). Humble leaders develop and promote not only high-quality leader-follower relationships but also helpful and supportive interpersonal relations (Ou et al., 2014). Indeed, leader humility has a contagion of a behavioral effect whereby employees can learn and follow the helping behaviors of a humble leader (Owens and Hekman, 2016). Qin et al. (2019) found a flow-on effect from the Chinese leader’s humility behaviors on their employees’ humility and subsequently, influence employees’ organizational citizenship behavior (OCB).

The literature addresses that knowledge sharing is a process of mutual exchange and creation of knowledge between individuals (Gagné, 2009). Thus, it is a form of OCB that requires individual willingness to cooperate and collaborate with others within an organization (Casimir et al., 2012). As knowledge is a personal asset, employees are willing to share knowledge when they intrinsically enjoy and derive pleasure from positive social exchange relationships with others (Kankanhalli et al., 2005; Anand et al., 2019). In this study, we are interested in the OCB-I as we aim to expand the emerging stream of research on the impacts of leader humility on the helping behavior of employees toward another employee (Carnevale et al., 2019) in the process of knowledge sharing. Accordingly, OCB-I includes a set of intentionally and discretionary helping behaviors of an employee toward others (Lee and Allen, 2002). Supervisors who are humble tend to exhibit behaviors that are necessary for generating collaboration and cooperation in the workplace (Ou et al., 2014; Owens and Hekman, 2016). Those behaviors would trigger down to their subordinates to strengthen employees’ sense of helping and exhibit OCB-I and assistance to others beyond their job descriptions (Owens et al., 2013; Ete et al., 2020) in response to a favorable exchange relationship with their supervisors (Cropanzano et al., 2017). Other empirical research from China (Carnevale et al., 2019) affirmed that when employees perceive their supervisors possess humility characteristics, they are likely to engage in OCB-I. Other scholars (Wasko and Faraj, 2005; Lin and Joe, 2012) also found that employees who engage in helping behaviors are likely to be intrinsically motivated toward knowledge sharing with their co-workers. Therefore, we expect OCB-I to be influenced by the supervisor’s humility behavior:

Hypothesis 2c: There is a positive association between leader humility and employees’ OCB-I.

### Affective Trust, Work Engagement, and OCB-I: Sequential Mediators

Drawing from SET, we hypothesize that three variables (affective trust, work engagement, and OCB-I) mediate the relationship between leader humility and knowledge sharing intention among employees. Specifically, employees develop trust in their leaders when they perceive trustworthy relationships and experience with the leaders (e.g., Rousseau et al., 1998). Gould-Williams (2003) argued that trust in leaders is the psychological and fundamental factor determining employees’ attitudes and behaviors that form high-quality interpersonal relationships, collaborations, and cooperation among organizational members (see Burke et al., 2007). From the SET perspective, employees’ trust in their supervisors stimulates work engagement as a personal obligation and reciprocity norms (Engelbrecht et al., 2017).

Oc et al. (2020) noted that leader humility behaviors (including a willingness to admit their mistakes and acknowledging their limitations combined with their tendency to appreciate the contributions of others) lead to an increase in trust in leaders. We argue that employees are likely to reciprocate their work engagement to a trusting relationship with humble leaders as leader humility behaviors signal the organization’s recognition of employees’ contributions (Owens et al., 2013; Argandona, 2015).

Although the relationship between work engagement and OCB-I has not received much attention, some scholars (Reijseger et al., 2017; Newton and LePine, 2018; Carnevale et al., 2019) implied a direct relationship between work engagement and OCB-I. Indeed, they argued that engaged employees are more likely to be more conscientious and willing to show OCB-I as good citizens of the organization who oblige the behavioral norms of a supportive work environment that a humble leader fosters. Research has found support for using SET to explain the positive association between work engagement and OCB when employees are in a respectful and trusting work relationship with their supervisors (Nguyen et al., 2019).

According to SET, employees feel obligated to perform OCB-I and care for the needs of others due to the influence of leader humility (Argandona, 2015; Owens and Hekman, 2016; Carnevale et al., 2019). When employees perform above and beyond their job descriptions, they are more likely to want to share knowledge with their work colleagues (Teh and Sun, 2012; Han et al., 2019). They are also unlikely to expect or consider any reciprocal benefit as an exchange for the trustworthy relationships established by their humble leaders (Carnevale et al., 2019). As a discretionary behavior, OCB-I has a direct impact on knowledge sharing among knowledge workers in countries such as Malaysia (Teh and Sun, 2012) and Korea (Jo and Joo, 2011).

Drawing from the previous discussion, we hypothesize the following:

Hypothesis 3a: The relationship between leader humility and knowledge sharing intention is mediated by affective trust in supervisor.
Hypothesis 3b: The relationship between leader humility and knowledge sharing intention is mediated by two serial mediators: affective trust in supervisor and work engagement.
Hypothesis 3c: The relationship between leader humility and knowledge sharing intention is mediated by three serial mediators: affective trust in supervisor, work engagement, and OCB-I.

**FIGURE 1:**
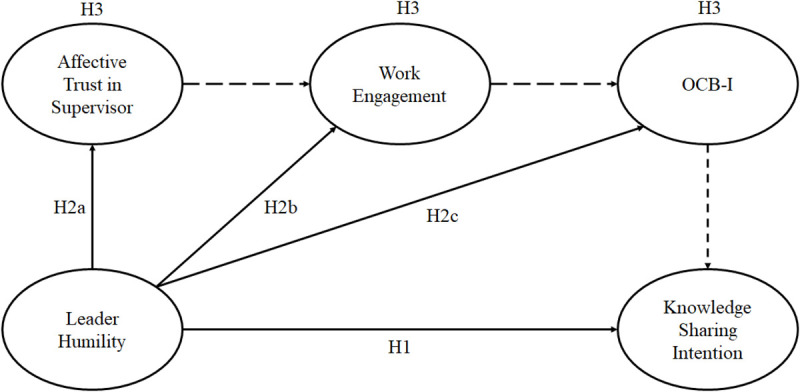
Proposed research model. Dotted lines indicated mediation hypotheses 3a, 3b, and 3c.

In summary, we adopt the SET perspective to develop the above hypotheses to test the effects of leader humility on knowledge sharing intention (see Figure 1).

## Materials and Methods

### Sampling Procedure and Demographic Background

Data were collected in 2019, with the assistance of an online research panel provider. This approach allowed us to access to reliable and valid data from professional workers (Brandon et al., 2013). Despite the drawbacks of a cross-sectional design, cross-sectional data is most relevant to establish the relationships between purported environments where the respondents are embedded (e.g., their relationship with supervisors), perceptual, and outcome variables (Spector, 2019). Furthermore, we collected cross-sectional data as we aimed to examine the naturally occurring effects of leader humility on other constructs in our model (Spector, 2019). Finally, a national-wide data collection process provides the possibility of the generalizability of the findings as we aimed to determine some foundational relationships between the study variables that have not been well-established in the literature (Spector, 2019).

We excluded irrelevant respondents who did not meet the inclusion criteria (i.e., full or part-time employment, residents of Australia, aged between 21 and 65 years old, and professional occupations). To minimize common method bias (Podsakoff et al., 2012), respondents who met the inclusion criteria, completed the online survey twice, separated by a four-week interval. This research design is consistent with other knowledge management studies (Škerlavaj et al., 2018; Feng and Wang, 2019).

At Time 1 (T1), the respondents provided information on their demographic background and the leader humility behaviors of their immediate supervisor. Affective trust, work engagement, OCB-I, and knowledge sharing were provided at Time 2 (T2). The final sample size after wave two data collection was 252 (a response rate of 55.75%). We have performed Soper’s (2020) *A priori* Sample Size Calculator for determining the power of SEM. With a medium effect size (Cohen’s *d* = 0.5), the desired statistical power level of 0.8, and probability level of 0.05, the calculation showed that the minimum total sample size for a two-tailed hypothesis is 128. The sample size of the current study was 252, which was greater than the minimum sample size recommended. This indicate adequate power and effect size to yield the flexibility and accuracy of the four-predictor model. To ensure the power and effect size, we further conducted a *post hoc* statistical power calculator for a student *t*-test with a medium effect size (Cohen’s *d* = 0.5), the desired statistical power level of 0.8, and probability level of 0.05. The result showed that the observed power for two-tailed hypothesis was 0.98, indicating an acceptable power and effect size.

Approximately two thirds of the respondents were female. Nearly half of the respondents (49.2%) were between 31 and 50 years old. More than two thirds of the participants were employed full-time. More than three quarters of the respondents were from the three largest states in Australia (New South Wales, Victoria, and Queensland). A large proportion of the respondents (72%) had at least three years of experience in their current position. More than half of the participants (54.7%) were degree graduates. More than half (58.4%) were from firms in knowledge-intensive service industries (such as health care and social assistance, scientific and technical services, education and training, and administrative support services).

### Measures

Previously validated scales were used in this study. Descriptive statistics and exploratory factor analysis (EFA) were conducted using IBM *SPSS version 25*. We then evaluated the convergent and discriminant validity of the latent variables and tested hypotheses using IBM *AMOS version 25*. Table 1 presents the measurement properties of the five latent variables and their item loadings.

**TABLE 1:** Measurement properties.

**Variable**	**Item loading**	**Composite reliability**	**AVE**
**Leader humility**		0.96	0.71
Your supervisor actively seeks feedback, even if it is critical	0.77		
Your supervisor admits it when he or she doesn’t know how to do something	0.82		
Your supervisor acknowledges when others have more knowledge and skills than himself or herself	0.81		
Your supervisor takes notice of others’ strengths	0.83		
Your supervisor often compliments others on their strengths	0.83		
Your supervisor shows appreciation for the unique contributions of others	0.88		
Your supervisor shows a willingness to learn from others	0.86		
Your supervisor shows he or she is open to the advice of others	0.88		
Your supervisor shows he or she is open to the ideas of others	0.9		
**Affective trust in supervisor**		0.92	0.7
We have a sharing relationship. We can both freely share our ideas, feelings, and hopes	0.76		
I can talk freely to this individual about difficulties I am having at work and know that (s)he will want to listen	0.84		
We would both feel a sense of loss if one of us was transferred and we could no longer work together	0.86		
If I shared my problems with this person, I know (s)he would respond constructively and caringly	0.93		
I would have to say that we have both made considerable emotional investments in our working relationship	0.76		
**Work engagement**		0.93	0.68
At my work, I feel bursting with energy	0.78		
At my job, I feel strong and vigorous	0.81		
I am enthusiastic about my job	0.9		
My job inspires me	0.92		
When I get up in the morning, I feel like going to work	0.79		
I am immersed in my work	0.73		
**OCB-I**		0.9	0.64
Willingly give your time to help others who have work-related problems	0.8		
Go out of the way to make newer employees feel welcome in the work group	0.76		
Show genuine concern and courtesy toward co-workers, even under the most trying business or personal situations	0.83		
Give up time to help others who have work or non-work problems	0.87		
Assist others with their duties	0.74		
**Knowledge sharing intention**		0.91	0.77
I will make an effort to share knowledge with my colleagues	0.84		
I intend to share knowledge with my colleagues when they ask	0.89		
I will share knowledge with my colleagues	0.89		

#### Leader Humility

We adopted a nine-item scale from Owens et al. (2013) to measure the respondents’ perceptions of their direct supervisor’ leader humility behaviors (α = 0.96; AVE = 0.71). These were rated from “1” = strongly disagree to “5” = strongly agree (sample items included “Your supervisor takes notice of others’ strengths”).

#### Affective Trust in Supervisor

We measured affective trust in the supervisor using the five-item scale from McAllister (1995) and adopted by Kim et al. (2016) (α = 0.92; AVE = 0.70). Respondents were asked to indicate their feeling toward their supervisor from “1” = strongly disagree to “5” = strongly agree (sample items included “We have a sharing relationship. We can both freely share our ideas, feelings, and hopes”).

#### Work Engagement

We used the nine-item Utrecht Work Engagement Scale (Schaufeli and Bakker, 2003) to measure work engagement. They responded to nine statements on a seven-point Likert scale anchored by “1” = strongly disagree to “7” = strongly agree. We removed threeitems due to their low factor loadings to increase the Cronbach’s alpha coefficient (α = 0.93; AVE = 0.68). Sample items included “At my work, I feel bursting with energy.”

#### OCB-I

An eight-item scale from Lee and Allen (2002) was used to measure the respondents’ frequency of performing citizenship behaviors toward co-workers. The items were rated using a scale ranging from “1” = never to “7” = always (sample items included “I willingly give my time to help others who have work-related problems”). We removed three items to improve the reliability and validity of the scale (α = 0.90; AVE = 0.64).

#### Knowledge Sharing Intention

We utilized a three-item scale from Ryu et al. (2003) to measure the respondents’ intended behaviors of knowledge sharing with others in organizations (α = 0.91; AVE = 0.77). The respondents were asked to think about interpersonal relationships at work and indicate the level of agreement or disagreement with the statements demonstrated their intention to share knowledge with others on a seven-point Likert scale from “1” = strongly disagree to “7” = strongly agree (sample items included “I intend to share my knowledge if they ask” and “I will share knowledge with my colleagues).

#### Control Variables

Several control variables from the literature incorporated (Owens et al., 2013; Qian et al., 2020). These included age, gender, career tenure, and tenure with the current organization, highest level of education, and ownership type.

## Data Analysis and Empirical Results

### Measurement Model Estimation

Data analyses were undertaken using IBM *AMOS version 25* to evaluate the convergent and discriminant validity of the latent measures. First, as reported in Table 1, the factor loadings of items on their constructs were above the cut-off values of 0.70 (Hair et al., 2010). Second, the measurement model of five latent variables demonstrated satisfactory fit to the data (Hu and Bentler, 1999; Williams et al., 2009). Goodness of fit indices were as follow: χ^2^[322] = 512.46, CFI = 0.97, TLI = 0.96, RMSEA = 0.05, SRMR = 0.05, PClose = 0.61, indicating that the model fits to the data (Hu and Bentler, 1999; Williams et al., 2009). We undertook a series of Chi-square difference tests to compare the fit of the hypothesized model with alternative models. The results of the comparison are reported in Table 2, indicating that the fit of the proposed five-factor model had the better fit.

**TABLE 2:** Comparison of fit of the hypothesized model with alternative models.

**Model**		**λ^2^**	**df**	**CFI**	**TLI**	**RMSEA**	**SRMR**	**Δλ^2^/df**
Model 1	Baseline model (Five-factor model)	512.46	322	0.97	0.96	0.05	0.05	–
Model 2	Four-Factor Model (OCB-I and Knowledge Sharing Intention were combined)	894.70	326	0.91	0.90	0.08	0.07	Δλ^2^(4) = 337.24, *p* < 0.001
Model 3	Three-Factor Model (OCB-I, Knowledge Sharing Intention, and Work Engagement were combined)	1,662.12	329	0.79	0.76	0.13	0.20	Δλ^2^(7) = 1,149.66, *p* < 0.001
Model 4	Two-factor model (OCB-I, Knowledge Sharing Intention, Work Engagement, and Affective Trust in Supervisor were combined)	2,062.78	331	0.73	0.69	0.14	0.15	Δλ^2^(9) = 1,550.32, *p* < 0.001
Model 5	Single factor model (Harman’s one factor model)	2,490.16	332	0.67	0.62	0.16	0.17	Δλ^2^(10) = 1,977.70, *p* < 0.001

As reported in Table 3, the values of composite reliability (CR) and average variance extracted (AVE) of the constructs were greater than the minimum cut-off values which suggest that the constructs had convergent validity (Hair et al., 2010). Additionally, the square root value of AVE of each construct was greater than its correlations with other variables. The AVE value of individual construct was larger than its relative MSV value (Hair et al., 2010). Heterotrait-monotrait ratio of correlations (HTMT)^1^ values between the five latent constructs were below 0.90 (Henseler et al., 2015). These tests allowed us to conclude that the five latent constructs had convergent and discriminant validity.

**TABLE 3:** Demographic statistics and correlations between variables.

**Variable**	**Mean**	**SD**	**1**	**2**	**3**	**4**	**5**	**6**
1. Age	4.48	1.41	1.00					
2. Gender	1.61	0.49	–0.10	1.00				
3. Position Tenure	3.34	1.30	0.38***	–0.08	1.00			
4. Organizational Tenure	3.47	1.24	0.39***	–0.08	0.74***	1.00		
5. Education	2.86	1.27	0.08	0.12	–0.04	–0.08	1.00	
6. Organizational Ownership	3.29	1.25	0.09	–0.09	0.09	0.01	–0.04	1.00
7. Leader Humility	3.50	0.93	−0.15*	–0.05	−0.15*	–0.10	–0.04	0.17**
8. Affective Trust in Supervisor	4.00	1.20	−0.14*	0.00	–0.02	0.03	–0.04	0.18**
9. Work Engagement	4.22	1.15	–0.02	–0.03	–0.01	0.08	0.02	–0.03
10. OCB-I	4.72	1.03	–0.04	0.19**	–0.03	0.04	0.12	−0.19**
11. Knowledge Sharing Intention	5.32	0.97	0.12	0.12*	–0.02	0.04	0.16*	–0.07

**Variable**	**CR**	**AVE**	**MSV**	**7**	**8**	**9**	**10**	**11**

7. Leader Humility	0.96	0.71	0.42	***0.84***				
8. Affective Trust in Supervisor	0.92	0.70	0.42	0.64***(0.64)	***0.83***			
9. Work Engagement	0.93	0.68	0.37	0.50***(0.51)	0.60***(0.63)	***0.82***		
10. OCB-I	0.90	0.64	0.26	0.05 (0.05)	0.19**(0.18)	0.27***(0.26)	***0.80***	
11. Knowledge Sharing Intention	0.91	0.77	0.42	0.17**(0.15)	0.13*(0.14)	0.24***(0.23)	0.51***(0.51)	***0.88***

*N* = 252. Bold and italic numbers are the square root values of AVE. Numbers in brackets are the HTMT values. Control variables: age ranges (six groups of age ranges); gender (0 = male, 1 = female); career tenure and tenure with the current organization (five groups of years of experience); education (1 = college degree, 2 = undergraduate degree, 3 = diploma, 4 = graduate degree including master and Ph.D. degrees, and 5 = others); organizational ownership (1 = public, 2 = non-profit, 3 = local government, 4 = private, and 5 = others). * *p* < 0.05, ** *p* < 0.01, *** *p* < 0.001.

### Common Method Variance

We undertook procedural and process remedies to minimize the effect of common method variance (CMV; see Podsakoff et al., 2012). As previously explained, a time lagged research design (separated by 4 weeks) was adopted. Participants were assured confidentiality and anonymity to minimize social desirability effect (Podsakoff et al., 2012). Different endpoint scales and random order of variables were used in the survey. A marker variable (“social desirability” scale) was included in the structural model (Lindell and Whitney, 2001). The differences of correlations of the five constructs before and after including the marker variable was 0.01 less than the cut-off value of 0.20. All these tests concluded that CMV was not a concern in our study.

### Hypothesis Testing

As presented in Table 3, the respondents in this study reported a moderate level of leader humility behaviors (Mean = 3.50, SD = 0.93) and a high level of intention to share knowledge (Mean = 5.32, SD = 0.97). The remaining three social exchange variables were rated at an average level: affective trust in supervisor (Mean = 4.00, SD = 1.20), work engagement (Mean = 4.22, SD = 1.15) and OCB-I (Mean = 4.72, SD = 1.03).

**FIGURE 2:**
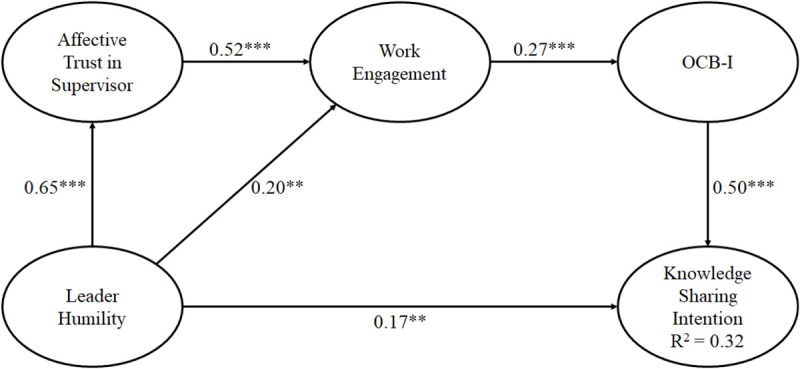
Significant results of direct relationships of proposed variables. Straight lines demonstrate direct effects. ^∗∗^*p* < 0.01, ^∗∗∗^*p* < 0.001.

The model (see Figure 2) had a goodness of fit (χ^2^[408] = 648.17, CFI = 0.96, TLI = 0.96, RMSEA = 0.05, SRMR = 0.05). Hypothesis 1 was supported as there was a positive association between leader humility behaviors and knowledge sharing intention (β = 0.17, *p* < 0.01). The relationship between leader humility and affective trust in supervisor was positive (β = 0.65, *p* < 0.001). Hypothesis 2a was supported. While there was a positive association between leader humility and work engagement (β = 0.20, *p* < 0.01), supporting Hypothesis 2b, Hypothesis 2c about the relationship between leader humility and OCB-I was not supported.

There was a direct, positive association between affective trust in supervisor and work engagement (β = 0.52, *p* < 0.001) and from work engagement to OCB-I (β = 0.27, *p* < 0.001). Finally, there was a direct, positive association between OCB-I and knowledge sharing intention (β = 0.50, *p* < 0.001). We performed the mediating hypothesis testing using the estimand plug-in by Gaskin (2016). These were “Specific Indirect Effects_Path” and Serial Mediation estimand, which were undertaken in IBM *AMOS version 25*. Mediation analysis (based on a 10,000 bootstrap samples) showed there was a partial, indirect relationship between perceived supervisor humility and knowledge sharing intention. There were two mediation relationships between leader humility and knowledge sharing intention. The first serial mediation was from leader humility → affective trust → work engagement → OCB-I → knowledge sharing intention [*b* = 0.10, SE = 0.03, 95% CI (0.05, 0.17), *p* < 0.01]. The second mediation was from leader humility → work engagement → OCB-I → knowledge sharing intention [*b* = 0.03, SE = 0.02, 95%CI (0.01, 0.07), *p* < 0.05]. These results supported Hypothesis 3c.

## Discussion

This study adopted SET to develop and test a model on how leader humility influences knowledge sharing intention from data of 252 employees from Australia. We identified a direct and positive association between leader humility and knowledge sharing intention. Also, the current study is one of the first to contribute to the leader humility and knowledge management literature by proposing and empirically finding the evidence of three serial exchange-based mediators, including affective trust, work engagement, and OCB-I.

### Theoretical Implications

Knowledge sharing is a vital mechanism by which transfer of knowledge can take place (Cabrera and Cabrera, 2005). Our findings provided empirical support to show the underlying organizational and motivational factors to encourage employees to share knowledge with their co-workers (Wang and Noe, 2010; Gagné et al., 2019). Scholars (Gagné, 2009; Wang and Noe, 2010) highlighted the role of leadership in making knowledge sharing happen within an organization. Extending the frameworks from Gagné (2009) and Wang and Noe (2010), our study highlights the significance of a high-quality reciprocal relationship with followers created by leader humility as a critical organizational factor in fostering knowledge sharing intention among employees. The literature portrays that humility is “one of the chief virtues in the business world” (Argandona, 2015, p. 63). Also, the exercise of humility is critical for the quality and effectiveness of leadership and enhances interpersonal relationships within an organization (Owens et al., 2013). Humble behaviors exhibited by leaders also facilitate the development of a positive and supportive work environment (Owens et al., 2013). Also, the qualities of leader humility highlight leaders’ recognition and appreciation of others’ strengths and vulnerabilities, the encouragement to cooperation and collaborations, and openness to new ideas (Owens et al., 2013). Drawing from the SET, we regard our findings by showing that employees are willing to reciprocate positive attitudes and behaviors that are proportional to the respectful and fair treatment from humble leaders by showing affective trust in supervisor, work engagement, OCB-I, and knowledge sharing intention.

By uncovering the black box in the relationship between leader humility and knowledge sharing intention, our study is among the first attempts to enrich our understanding of the specific effects of leader humility on knowledge sharing intention through a lens of SET. Our study first provides initial evidence that leader humility directly makes employees feel supported to contribute new ideas and exchange information with others in cooperation and collaboration. This direct relationship occurs as a humble leader encourages followers to admit mistakes, take accountability for actions, and listen to different and opposing opinions in a non-judgmental manner (Owens et al., 2013; Argandona, 2015). Based on this finding, we contribute to the SET literature that employees’ knowledge sharing intention could be a form of the reciprocal norm.

The second finding in our study posits that employees enjoy a trustworthy relationship with a humble supervisor. The reciprocation of affective trust in a supervisor occurs when a humble supervisor is a role model who exercises honesty, fairness, responsibility, empathy, and care for subordinates to inspire and create a high level of emotional bonds between the supervisor and employees (Owens et al., 2013; Argandona, 2015). From a SET perspective, our results highlight that employees will reciprocate work engagement to a trustworthy and supportive relationship with humble leaders. These findings indicate that employees’ affective trust in a supervisor and work engagement is the most critical mechanisms of a social exchange process (Owens et al., 2013).

Although we expected the direct relationship between leader humility and OCB-I (Hypothesis 2c), we could not find the support for this hypothesis. Instead, our finding shows that OCB-I is a SET-outcome of affective trust and work engagement. Not surprisingly, this finding remains supporting SET propositions such that employees could perceive OCB-I as a norm of reciprocity in an exchange relationship with the leader just when they trust the leader that increases their engagement at work. Our study is different from Ete et al. (2020) by showing supporting evidence for the mediating effects of affective trust and work engagement. Our study represents a novel contribution to the literature of leader humility and OCB by showing the boundary conditions of the effectiveness of leader humility on employee OCB-I.

The last but not least contribution of our study to the literature on knowledge management is the mediating mechanism in which how leader humility encourages followers to share knowledge. Support for our serial mediator model depicts the unique contribution in showing that the motivation derived from the social exchange with one’s immediate supervisor who exhibits humility behaviors is positively associated with affect trust in the supervisor (Wang et al., 2019; Yang et al., 2019). This positive association will then be reciprocated with enhanced levels of work engagement (Nielsen and Marrone, 2018). When employees are engaged, they experience the associated positive energy derived from being treated well by their immediate supervisors (Kim et al., 2015) that foster the willingness to help their co-workers (Bartol et al., 2009). These attitudinal and behavioral mediators are fundamental for nurturing the relationship between leadership and employees’ knowledge sharing intention. Our study consequently provides further support for the application of SET in linking leader humility with the knowledge sharing literature (Serenko and Bontis, 2016).

### Managerial Implications

Knowledge sharing within organizations is vital in today’s increasingly global economies. Our study proposes a SET perspective that highlights how leader humility can, directly and indirectly, influence knowledge sharing intention among employees. Accordingly, a subordinate’s positive perception of the humility of their immediate supervisor contributes to the creation of a trusting and supportive work environment that is conducive to knowledge sharing. Based on this finding, organizations need to be mindful of the personality characteristics and behavioral exhibition of humble leaders. These behaviors can help shape a cooperative, collaborative, and supportive environment to cultivate knowledge sharing. We also suggest Human Resource (HR) managers focus on using HR practices to recruit, select, retain, and develop supervisors to exhibit leader humility behaviors. For example, when selecting individuals for managerial/supervisory positions, HR managers could use tests to examine the humble behaviors and attitudes of leaders in how they treat their employees. More importantly, we recommend the emphasis on how humble leaders can communicate, engage employees, and promote knowledge sharing. Also, organizations could highlight the qualities of leader humility in performance expectations as part of their performance management processes as humble leaders lead by the examples and model of the attitudes and behaviors. Another important practical implication of our study is related to the mediating mechanism of three intervening variables (specifically, trust in supervisor, work engagement, and OCB-I) as critical outcomes of leader humility. These findings highlight that organizations need to pay attention to measuring the impacts of leader humility on these behaviors among employees. As trust is essential in knowledge sharing, humble leaders build employee trust as a reciprocal outcome of the leaders’ trust in people. Working with a trustworthy relationship with a humble leader, employees feel more engaged in their work, more obliged to be a good citizen in helping others. Overall, our findings help organizations to understand the implications of humble leader behaviors on knowledge sharing intention of employees. Despite differing knowledge management practices adopted by organizations to promote knowledge sharing, leader humility requires attention because of its profound consequences on the knowledge sharing intention of employees.

### Limitations and Future Research Implications

As previously indicated, this study aims to test a model that has the generalizability of the hypothesized model by utilizing a SET perspective. Theoretically, future research should extend the current SET perspective to incorporate dimensions of affect into the theorizing of knowledge sharing intention (Serenko and Bontis, 2016). As proposed in Lawler’s (2006) affect theory of social exchange, affect (or emotions) could be incorporated into a social exchange relationship as a social exchange at the workplace do result in emotional responses. A leader’s humility in relating to employees will result in positive emotions that could generate affective trust in the leader, which finally leads to employees’ willingness to share knowledge with their co-workers. This notion would extend the theoretical underpinning of Blau’s SET (Cropanzano and Mitchell, 2005). Also, future studies could consider other work-related variables as outcomes of leader humility, such as job control and autonomy, leader-member exchange, perceived organizational support, or supervisory support, in examining the mechanisms underlying the relationship between leader humility and knowledge sharing behaviors of employees.

We are aware of the potential bias due to the cross-sectional and single-source data. We followed the recommendations in the literature to implement procedural and statistical remedial checks (see Podsakoff et al., 2012) that provided further assurance that CMV was not an issue. Future studies could collect data from multiple sources (e.g., supervisor rating of focal respondents’ attitudes and behaviors). We also acknowledge the limitation of the cross-sectional design that could infer the testing of causal relationships between the variables. This notion requires the use of a longitudinal research design (Owens et al., 2013). Another recommendation is to use an experimental design (Škerlavaj et al., 2018).

Future studies could focus on leader humility and knowledge management across different national and cultural contexts. We noted that much research on leader humility has been in Asian societies with high power distance and collectivism. We encourage scholars to study the phenomenon in different contexts to these to better tease out the moderating effects of cultural values on leadership and knowledge sharing behaviors (Oc et al., 2015). Finally, in addition to varying the national context, it might also be worth investigating specific knowledge-based industries such as architecture or software designers as these were high knowledge-based professional workers.

## Conclusion

In summary, this study contributes to the literature on leadership and knowledge sharing by using a SET perspective. We provide new theoretical and empirical insights into the understanding of how leader humility can facilitate employees’ knowledge sharing. Our study shows that humble leaders role model supportive behaviors, directly and indirectly, through affective trust, work engagement, and OCB-I, nurtures employees’ intention to share knowledge. In so doing, our research contributes insights into the processes by which leadership, through reciprocal attitudes and behaviors, promotes knowledge sharing motivation.

## Data Availability Statement

The raw data supporting the conclusions of this article will be made available by the authors, without undue reservation.

## Ethics Statement

The studies involving human participants were reviewed and approved by Edith Cowan University Human Research Ethics Committee. The patients/participants provided their written informed consent to participate in this study.

## Author Contributions

DN and ST: project design, administration, formal analysis, and data curation. DN and ST: writing – original draft preparation. All authors: writing – review and editing.

## Conflict of Interest

The authors declare that the research was conducted in the absence of any commercial or financial relationships that could be construed as a potential conflict of interest.

## Footnotes

1 HTMT shows the average of the correlations of indicators across constructs relative to the average of the correlations of indicators within the same construct. This has been recommended as an alternative and superior approach to check the discriminant validity of constructs (Henseler et al., 2015).
